# Electroacupuncture Suppressed Neuronal Apoptosis and Improved Cognitive Impairment in the AD Model Rats Possibly via Downregulation of Notch Signaling Pathway

**DOI:** 10.1155/2015/393569

**Published:** 2015-02-25

**Authors:** Hai-dong Guo, Jin-xin Tian, Jing Zhu, Li Li, Kui Sun, Shui-jin Shao, Guo-hong Cui

**Affiliations:** ^1^Department of Anatomy, School of Basic Medicine, Shanghai University of Traditional Chinese Medicine, Shanghai 201203, China; ^2^Department of Endocrinology, The People's Hospital of Zhangqiu, Shandong 250200, China; ^3^Department of Cardiology, The People's Hospital of Zhangqiu, Shandong 250200, China; ^4^Department of Neurology, Shanghai No. 9 People's Hospital, Shanghai Jiaotong University School of Medicine, Shanghai 200011, China

## Abstract

Acupuncture is a potential strategy for the treatment of Alzheimer's disease (AD) and the possible mechanisms worth to be explored. In this study, we proposed and tested the hypothesis that whether Notch signaling pathway is involved in the effect of electroacupuncture (EA) treatment. Rats that received EA treatment on the acupoints of Baihui (Du 20) and Shenshu (BL 23) had shorter latency and remained in the original platform quadrant longer and crossed the former platform contained quadrant more frequently compared to the A*β* injection rats without EA treatment. EA obviously alleviated the cell apoptosis resulted by A*β* infusion in hippocampus CA1 regions through upregulating the expression of Bcl-2 and downregulating the expression of Bax. EA could further obviously promote the expression of synapsin-1 and synaptophysin in hippocampus. A*β* injection significantly increased the expression of Notch1, Jag1, and Hes1 mRNA, while EA treatment downregulated the level of Notch1 and Hes1 mRNA in hippocampus, but not Jag1 mRNA. Our data suggested that EA treatment improved learning and memory function in the AD rat model partially through downregulating Notch signaling pathway.

## 1. Introduction

Alzheimer's disease (AD), the most common cause of dementia in the elderly, is a pernicious neurodegenerative disorder associated with cognitive and behavioral dysfunction. AD is characterized by the presence of neurofibrillary tangles and insoluble *β*-amyloid (A*β*) plaques that are associated with inflammation response and neuronal and synaptic loss [1]. Amyloidogenesis, neuroinflammation, and memory impairment through the injection of A*β* have been widely investigated [2, 3]. Up to date, only a few AD medications have been proved as improving AD symptoms, but they offer only marginal benefits [4] and do not compensate for the massive and progressive neuronal and synaptic loss in the cortex and hippocampus [5]. The increasing number of patients suffering from AD throughout the world indicates an urgent need for preventive measures and effective therapy [6].

Acupoints are considered to be located where nerves enter a muscle, the midpoint of the muscle, or at the enthesis where the muscle joins with the bone. Acupuncture points may exhibit low electrical resistance and impedance. Experimental studies have shown that acupuncture provides neuroprotection with antioxidation and antiapoptosis effects through stimulating the specific acupoints [7–9]. However, only a few acupuncture clinical studies with a small number of participants are reported. Acupuncture could enhance the hippocampal connectivity [10], activate certain cognitive-related regions in AD patients [11–13], and has potential therapeutic effects on improving cognitive function and self-managing ability of vascular dementia [14]. Electrical stimulation of acupuncture points, or electroacupuncture (EA), manipulated by passing electric currents through acupuncture needles, is also widely used and allows a more objective control over stimulating parameters. Although studies demonstrated that EA could improve behavioral performance [15, 16], the function is still controversial and its underlying mechanisms still remain elusive.

Signal transduction occurs when an extracellular signaling molecule activates a specific receptor located on the cell surface or inside the cell. After that, this receptor triggers a biochemical chain of events inside the cell, creating one or more responses, such as metabolism, shape, gene expression, or ability to divide. The canonical Notch signaling pathway is a highly conserved signal transduction pathway, which is fundamental for neuronal development and specification through regulating transcription of the Notch target genes, such as Hes1 and Hes5 [17]. Accumulating evidence indicates that canonical Notch pathway has often been implicated in AD; however, the mechanisms involving Notch in progressive neurodegeneration remain unclear [18]. EA pretreatment could induce the tolerance against focal cerebral ischemia through activation of canonical Notch pathway [19]. A recently study found that EA enhanced the proliferation of hippocampal neural stem cells in cerebral ischemia-reperfusion injured rats via regulation of Notch signaling pathway [20].

In the present investigation, we aimed to examine whether EA could affect A*β*-induced apoptosis, synaptic degeneration, and learning and memory deficits in A*β*-injected rats. Additionally, the levels of Notch signaling related genes, such as Notch1, Jag1, and Hes1 were measured to test whether Notch signaling pathway is involved in the effect of EA treatment on AD rats.

## 2. Materials and Methods

### 2.1. Intrahippocampally Injected A*β*_1–40_ Rat Model

A*β*_1–40_ (Sigma) was firstly dissolved in distilled water and then diluted in phosphate buffer saline (PBS) at a concentration of 2.5 *μ*g/*μ*L and incubated for 5 days at 37°C to make the state of aggregation prior to injection. Adult male Sprague-Dawley (SD) rats (250~300 g) were randomly assigned to four groups: control, model, EA, and sham-EA. All of the rats were anesthetized by intraperitoneal injection of sodium pentobarbital (50 mg/kg) and placed on a stereotaxic instrument (David Kopf Instruments, USA). The rats were administered 2 *μ*L of normal saline (control group) or A*β*_1–40_ (other groups) bilaterally in the hippocampus over 5 min using a Hamilton microsyringe with a 26S gauge needle at 3.8 mm posterior to bregma, ±3.2 mm lateral to midline, and 2.7 mm below dura, according to a stereotaxic atlas of the rat brain. The needle was left in place for an additional 5 min to allow diffusion into the surrounding tissue before slowly retracted. The experimental protocols were approved by the Ethics Committee for Animal Experimentation and were performed according to the Guidelines for Animal Experimentation of Shanghai University of Traditional Chinese Medicine.

### 2.2. EA and Sham-EA Treatment

Seven days after surgery, the acupoints of Baihui (Du 20) and Shenshu (BL 23) were stimulated once daily by an electroacupuncture apparatus (Model G-6805-2, Shanghai Medical Electronic Apparatus, China) in the EA group. The locations of the acupoints have been described previously [21, 22]. Stimulation was applied to the pair of acupoints with continuous wave (20 Hz) for 30 min per day. The intensity was adjusted to induce slight muscle contract of the hindlimb (≤2 mA). For the sham-EA group, nonacupuncture points sited at approximately 3 mm to the lateral side of the tail on the gluteus muscle were needled to observe the specificity of the acupoint effects. The rats in the other groups were grasped in the same amount of time and with the same extent of strength as that in the EA group. The treatment process was continued for 28 days with a rest every 7 days.

### 2.3. Morris Water Maze Test

Cognitive function was tested by the water maze, which was a circular pool with a diameter of 120 cm and a height of 50 cm. The pool was filled to a depth of 40 cm with water (22 ± 1°C) and divided into four equal quadrants. A circular escape platform (10 cm in diameter) was placed at the midpoint of the target quadrant and submerged approximately 1.5 cm below the surface of the water. For the place navigation trials, rats were trained for 5 days. Each trial was started by placing the rats in one of the four quadrants. Animals were allowed to swim in pool during a period of 70 s to find the hidden platform. If an animal did not find the platform within this period, it was manually guided to platform by researchers. The rats rested 30 s between two consecutive trials. Posttraining probe trial tests were conducted 1 day after the final training session. The hidden platform was removed, and rats were allowed to swim freely for 70 s. Then the occupancy and crossing of animals in proximity of target quadrant (the quadrant included hidden platform during training trials) were recorded.

### 2.4. Immunofluorescence and Hoechst 33342 Staining

Half of the rats per group were anesthetized by intraperitoneal injection of sodium pentobarbital immediately after Morris water maze test. Immunofluorescence staining for synapsin-1 was carried out on 6 mm thick coronal sections, made through the hippocampus of paraffin embedded brain. Sections were deparaffinized and rehydrated and antigen retrieval was performed by microwaving in citrate buffer. The sections were incubated with 5% normal serum in PBS for 30 min and were incubated overnight with rabbit antisynapsin-1 (1 : 100; Cell Signaling Technology, USA). After that, the sections were incubated with FITC-conjugated goat anti-rabbit IgG (Jackson ImmunoResearch, USA) for 60 min at room temperature and counterstained with DAPI (Sigma) and then observed with fluorescence microscopy.

Hoechst 33342 was used to assess the nuclear morphology of the neurons. Five sections per group (one section each animal) were utilized for quantitative analysis. The nuclear condensed cells in five random fields (200x) of hippocampus CA1 in each section were counted to indicate neuronal apoptosis.

### 2.5. Western Blot

Another half of animals were killed by rapid decapitation and the hippocampus was dissected; the left hippocampus was homogenized with ice-cold radioimmunoprecipitation assay (RIPA) lysis buffer containing protease inhibitor phenylmethylsulfonylfluoride (PMSF) with a glass homogenizer on ice. Samples containing 40 *μ*g of protein were boiled in SDS mercaptoethanol sample loading buffer, separated by 10% or 12% SDS-PAGE gel, and electrically transferred to PVDF membrane (Millipore, USA). Nonspecific binding was blocked by incubation of the membrane in 5% skimmed milk for 1 h. The PVDF membrane was then incubated overnight at 4°C with anti-Bcl-2 (1 : 2000; Abcam, UK), anti-Bax (1 : 1000; Abcam, UK), anti-synapsin-1 (1 : 1000; Cell Signaling Technology), or antisynaptophysin (1 : 1000; Cell Signaling Technology). Bound primary antibody was detected with HRP-conjugated anti-rabbit antibody (1 : 10000; Jackson ImmunoResearch) and blots were developed using an enhanced chemiluminescence detection system (ECL kit). The density of the specific bands was quantified with Image J software and normalized to GAPDH.

### 2.6. Real-Time PCR

The right hippocampus from another half of rats was harvested on ice and total RNA was isolated with Trizol reagent according to a standard protocol. A total of 1 *μ*g of each template RNA was converted to the first strand of cDNA. Real-time PCR of cDNA was performed with LightCycler 2 system (Roche, Switzerland) using SYBR Green ready mix (Applied Biosystems, USA) and the forward and reverse primer. The primers for each gene were Notch1: (Fwd: AATGGAGGGAGG TGCGAAG, Rev: ATGGTGTGCTGAGGCAAGG); Jag1: (Fwd: CCAGCGGTCCTAATGGTGATG, Rev: GCTGTGGTTCTGAGCTGCAAAG); Hes1: (Fwd: TGTCAACACGACACCGGACA, Rev: GCCTCTTCTCCATGATAGGCTTTG); GAPDH: (Fwd: GGCACAGTCAAGGCTGAGAATG, Rev: ATGGTGGTGAAGACGCCAGTA). The relative amount of each mRNA was normalized to the housekeeping gene GAPDH mRNA. The relative gene expression levels were calculated with the 2^−ΔΔCt^ method.

### 2.7. Statistical Analysis

The software, SPSS 11.5 for Windows (SPSS Inc., IL), was used to conduct statistical analyses. All values are presented as mean ± SD. To analyze the data statistically, one-way analysis of variance (ANOVA) with Student-Newman-Keuls post hoc multiple-comparison analysis was performed. Values of *P* < 0.05 were considered statistically significant.

## 3. Results

### 3.1. EA Rescued Behavioral Impairment of AD Rats

**Figure 1:**
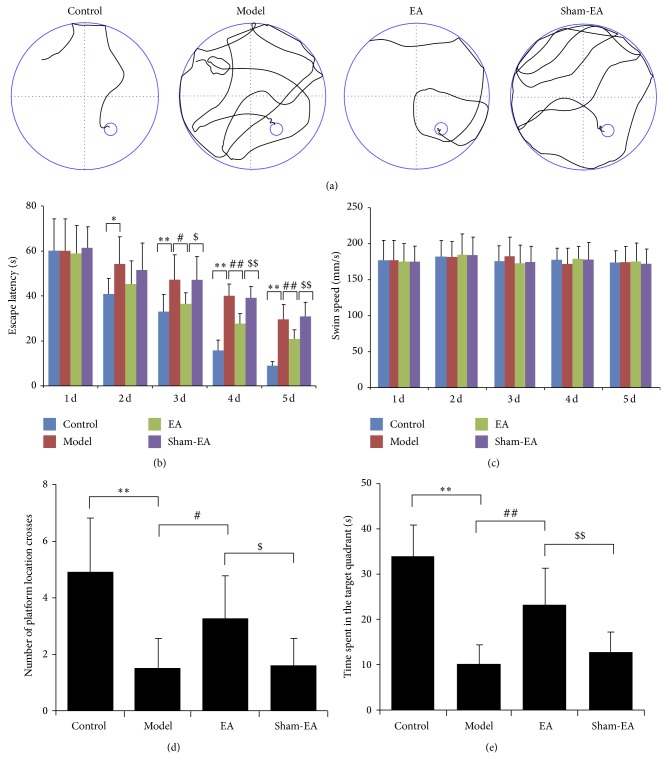
EA restored learning and memory in A*β*_1–40_-infused rat model. (a) Representative navigation traces of each group performing the Morris water maze task on day 5. (b) Escape latencies per day as assessed by the Morris water maze task. Improvement of cognitive function was only observed in the EA group and not in the sham-EA group. (c) Swimming speed was not significantly different among groups. (d) Probe analysis was performed on day 6. EA treated rats performed significantly better than the model and sham-EA treated rats based on the number of platform location crosses. (e) Rats received EA treatment remained in the original platform quadrant longer compared to model and sham-EA treated rats. ^*^ *P* < 0.05 and ^**^ *P* < 0.01 versus control, ^#^ *P* < 0.05 and ^##^ *P* < 0.01 versus model, and ^$^ *P* < 0.05 and ^$$^ *P* < 0.01 versus sham-EA.

The effects of EA on learning and memory ability in A*β*_1–40_ infused rats were assessed using Morris water maze test. In the place navigation trials, representative navigation paths at day 5 of training provided evidence that spatial learning acquisition was dramatically impaired in AD model rats but not in EA treatment ones (Figure 1(a)). The escape latencies in A*β*_1–40_ injected rats were significantly higher than those in control group in each same day from days 2 (*P* < 0.05 and *P* < 0.01). Animals in the group of EA treatment displayed obviously shorter escape latencies on days 3~5 of training than those of the model rats (*P* < 0.05 and *P* < 0.01), indicating improvements in spatial acquisition. The escape latencies in sham-EA group exhibited a similar pattern to the model group (*P* > 0.05) and there was a significant difference between the groups of EA and sham-EA in escape latency in days 3~5 (*P* < 0.05 and *P* < 0.01; Figure 1(b)). Swimming speed did not reveal any significant alterations among the four groups during the 5 training days (*P* > 0.05; Figure 1(c)). In the spatial probe trials, the number of platform location crosses and time spent in the target quadrant were much lower in model group compared with control group (*P* < 0.01). Although sham-EA treatment showed a trend of improvement in behavior recovery, no apparent difference was observed between the groups of model and sham-EA (*P* > 0.05). Interestingly, rats received EA treatment crossed the former quadrant containing the platform more frequently and remained in the original platform quadrant longer compared to rats of model group and sham-EA group (*P* < 0.05 and *P* < 0.01), suggesting impaired spatial memory recall after EA treatment (Figures 1(d) and 1(e)).

### 3.2. EA Attenuated the Neuronal Apoptosis Induced by A*β*

**Figure 2:**
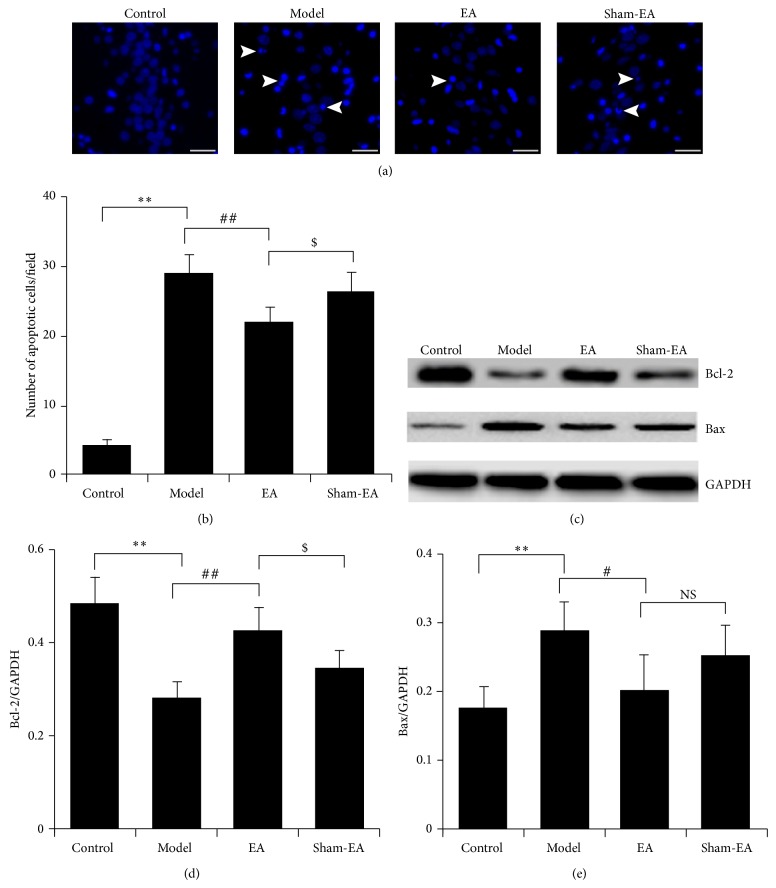
EA prevented the neuronal apoptosis induced by A*β* in hippocampus tissue through regulating the expression of Bcl-2 and Bax. (a) Neuronal apoptosis in CA1 region was detected by Hoechst 33342 staining. White arrowheads showed Hoechst 33342 positive apoptotic cells. Scale bar: 30 *μ*m. (b) Quantitative analysis of Hoechst 33342 positive cells in CA1 region under 200x fields. (c) Expression of Bcl-2 and Bax in hippocampus tissues examined by Western blot. ((d), (e)) Quantitative analysis of the expression of Bcl-2 and Bax normalized with GAPDH using data obtained from the different blots. ^**^ *P* < 0.01 versus control, ^#^ *P* < 0.05 and ^##^ *P* < 0.01 versus model, and ^$^ *P* < 0.05 versus sham-EA.

Hoechst 33342 staining was performed to investigate whether EA was able to improve behavioral impairment by preventing neuronal apoptosis. The results showed that the number of cells with condensed and fragmented DNA (apoptotic cells) significantly increased after A*β* injection compared to the control group (*P* < 0.01), while EA obviously alleviated the cell apoptosis resulted by A*β* infusion in hippocampus CA1 regions (*P* < 0.01). The number of apoptotic cells in EA group was much less than that in sham-EA group (*P* < 0.05; Figures 2(a) and 2(b)). Moreover, the expression of prosurvival protein Bcl-2 and proapoptotic protein Bax, well known to be involved in the canonical mitochondrial apoptotic pathway, were determined by Western blot in hippocampus tissues (Figure 2(c)). Samples from A*β* injected rats represented much lower level of Bcl-2 and higher level of Bax than in the control group (*P* < 0.01). EA could upregulate the expression of Bcl-2 and downregulate the expression of Bax compared with the model group (*P* < 0.01 and *P* < 0.05). Expression of Bcl-2 showed significant difference between the groups of EA and sham-EA (*P* < 0.05), while there was no statistic difference in the expression of Bax (*P* > 0.05; Figures 2(d) and 2(e)). These data indicated that EA could balance the expression of apoptosis related proteins Bcl-2 and Bax and prevent the neuronal apoptosis induced by A*β* in hippocampus.

### 3.3. EA Was Benefit to the Recovery of Synaptic Function

**Figure 3:**
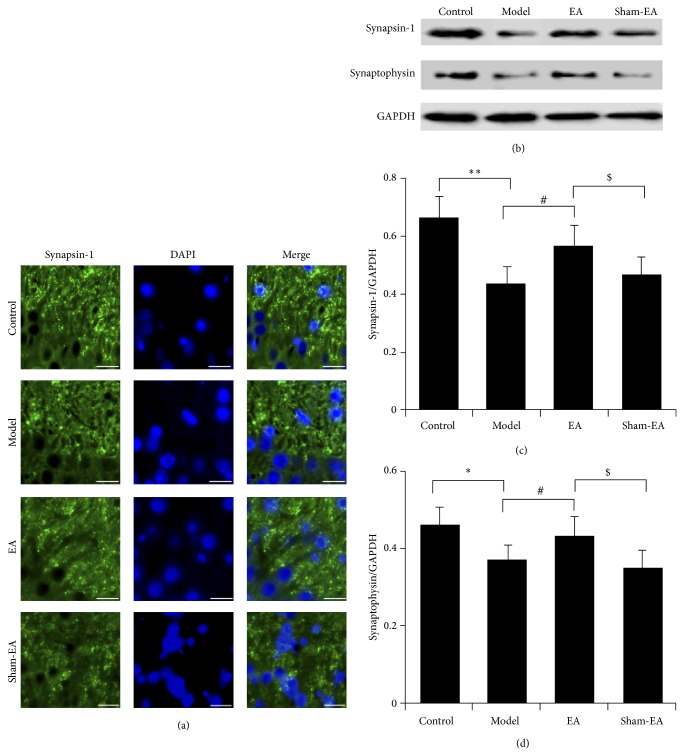
EA was benefit to the recovery of synaptic function. (a) Immunohistochemical analysis of synapsin-1 in CA1 region. Scale bar: 20 *μ*m. (b) Expression of synapsin-1 and synaptophysin was detected by Western blot. ((c), (d)) Quantitative analysis of the expression of synapsin-1 and synaptophysin. ^*^ *P* < 0.05 and ^**^ *P* < 0.01 versus control, ^#^ *P* < 0.05 versus model, and ^$^ *P* < 0.05 versus sham-EA.

Synaptic degeneration is another ultimate event of AD except neuronal loss and synaptic proteins are essential components to maintain normal synaptic function. Therefore, we next determined the expression of presynaptic protein synapsin-1 and synaptophysin in hippocampus. Data from both immunohistochemistry staining and Western blot showed that the level of synapsin-1 was markedly decreased owing to A*β* injection (*P* < 0.01). However, the expression of synapsin-1 in the hippocampus was significantly elevated in rats received EA treatment, compared with the model group and sham-EA group (*P* < 0.05; Figures 3(a) and 3(b)). Similarly, the level of synaptophysin in A*β* injected rats was much higher than that in control group (*P* < 0.05) and EA effectively increased the expression of synaptophysin compared to both model and sham-EA groups (*P* < 0.05; Figures 3(b) and 3(c)).

### 3.4. EA Downregulated Notch Signaling Pathway in Rat Hippocampus

**Figure 4:**
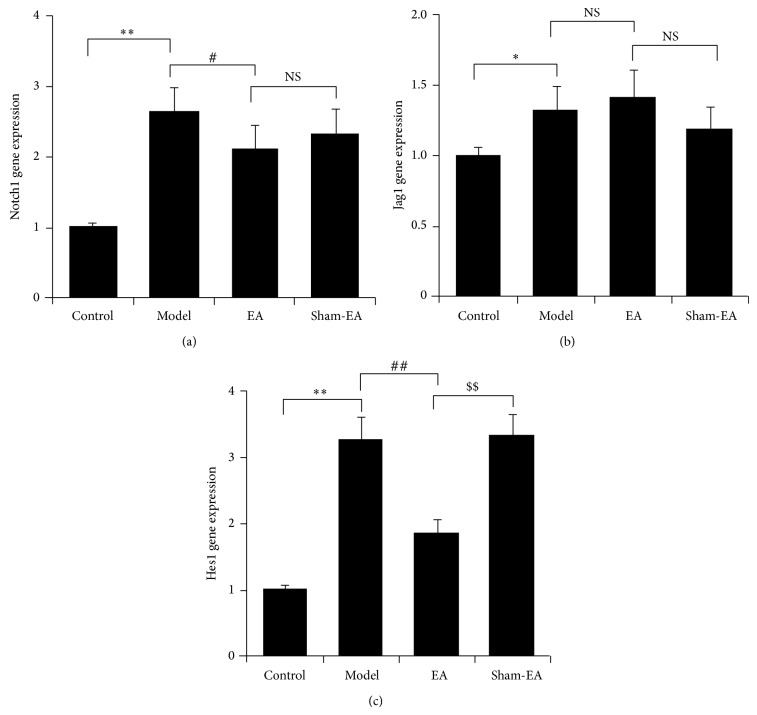
Downregulation of the Notch signaling pathway in rat hippocampus after EA treatment. Quantitative real-time PCR of mRNA expression of Notch1 (a), Jag1 (b), and Hes1 (c) in each group. ^*^ *P* < 0.05 and ^**^ *P* < 0.01 versus control, ^#^ *P* < 0.05 and ^##^ *P* < 0.01 versus model, and ^$$^ *P* < 0.01 versus sham-EA.

To investigate the mechanisms underlying the beneficial activity of EA, real-time PCR analysis for Notch1, Jag1, and Hes1 genes, which are major Notch pathway components in the central nervous system, was performed. A*β* injection obviously stimulated the expression of Notch1, Jag1, and Hes1 mRNA (*P* < 0.05 and *P* < 0.01). The Notch1 and Hes1 mRNA in hippocampus in EA group was downregulated compared with model group (*P* < 0.05 and *P* < 0.01). There was no significant difference in Jag1 mRNA level between EA and model groups (*P* > 0.05). Compared to the sham-EA group, rats from EA group showed a much lower level of Hes1 mRNA (*P* < 0.01; Figures 4(a)–4(c)).

## 4. Discussion

Acupuncture is one of the most promising supplementary medical treatments because of easy application, low cost, and minimum side effects. Although acupuncture is a potential intervention for the treatment of AD [14–16], the effect is still controversial [23]. In this study, we selected acupoints Baihui (Du 20) and Shenshu (BL 23), which directly communicate with or nourish the brain, to verify the effectiveness of EA for AD [24]. We found that a single infusion of A*β*_1–40_ effectively impaired learning and memory behavior. Consistent with a series of previous studies, the present study confirmed that rats received EA treatment had much shorter latency and remained in the original platform quadrant longer and crossed the former platform contained quadrant more frequently compared to the A*β* injection rats without EA treatment. The behavior function in sham-EA group exhibited a similar pattern to the model group and much worse than that in EA group, indicating that acupoint specificity on learning and memory recovery effect of EA.

Neuronal loss is the ultimate event and the main cause of irreversible progression of AD. Reducing or inhibiting the level of apoptosis of hippocampal neurons in acute AD would be the key strategy to restore the learning and remember function. Bcl-2 family proteins, the essential regulator of cell apoptosis, are the main pathways involved in A*β*-induced cell death. In the present study, we found that, after injection of A*β*, many of cells in CA1 region appeared obvious apoptotic morphology with dramatically decreased level of Bcl-2 and increased level of Bax. EA treatment could decrease the number of apoptotic cells induced by A*β* in hippocampal CA1 region through balancing the expression of apoptosis related proteins Bcl-2 and Bax.

In the nervous system, synapses are essential to neuronal function and the synapse is a structure that permits a neuron to pass an electrical or chemical signal to another cell. Reduction in the number of synapses has been reported in normal aging human subjects and AD patients [25]. Synapsin-1 and synaptophysin are presynaptic proteins which regulate neurotransmitter release and efficiency of the synaptic vesicle cycle [26, 27]. EA at the Baihui (Du 20) and Dazhui (GV14) acupoints could exert beneficial effects on synaptic reconstruction [24]. Our data showed that EA, not sham-EA treatment, could effectively alleviate the downregulation of synapsin-1 and synaptophysin induced by A*β* injection. The data indicated that EA not only attenuated the apoptosis of hippocampal neurons but also could be benefit to the recovery of synaptic function. However, effect of EA on the expression of postsynaptic protein, such as PSD95, needed further to be disclosed.

To better understand the effect of EA on learning and memory recovery, we moved forward to test the expression of Notch signaling pathway related genes for the first time. Notch pathway appears to be involved in neural progenitor regulation, neuronal connectivity, synaptic plasticity, and learning/memory [28]. We found that A*β* injection significantly stimulated the expression of Notch1, Jag1, and Hes1 mRNA, while EA treatment obviously downregulated the level of Notch1 and Hes1 mRNA in hippocampus after A*β* injection. Expression of the Notch effector gene Hes1 is required for maintenance of neural progenitors in the embryonic brain, but persistent and high levels of Hes1 expression inhibit proliferation and differentiation of neural progenitors. Thus, the regeneration of neurons from neural progenitors may be impaired due to the abnormal elevated Notch signal pathway. Numerous microRNAs (miRNAs) were downregulated in response to A*β*, including miRNA-9, a synapse-enriched miRNA that is decreased in Alzheimer's disease. It has been found that miRNA-9 attenuated A*β*-induced synaptotoxicity by targeting CAMKK2 [29]. Interestingly, Hes1 has been identified as a direct target of miRNA-9 [30]. It is well known that one miRNA can target several genes, while one gene may be regulated by multiple miRNAs. Therefore, it is very meaningful and valuable to explore the effect of EA on the changes of miRNA network to further reveal the molecular mechanism and therapy targets of AD.

## 5. Conclusion

In conclusion, results from the present study suggested that EA treatment reduced neuronal apoptosis and synaptic degeneration and enhanced learning and memory recovery in the AD model rats possibly through downregulating Notch signaling pathway.
